# The Role of Particulate Matter-Associated Zinc in Cardiac Injury in Rats

**DOI:** 10.1289/ehp.10379

**Published:** 2007-10-24

**Authors:** Urmila P. Kodavanti, Mette C. Schladweiler, Peter S. Gilmour, J. Grace Wallenborn, Bhaskar S. Mandavilli, Allen D. Ledbetter, David C. Christiani, Marschall S. Runge, Edward D. Karoly, Daniel L. Costa, Shyamal Peddada, Richard Jaskot, Judy H. Richards, Ronald Thomas, Nageswara R. Madamanchi, Abraham Nyska

**Affiliations:** 1 National Health and Environmental Effects Research Laboratory, Office of Research and Development (ORD), U.S. Environmental Protection Agency, Research Triangle Park, North Carolina, USA; 2 Center for Environmental Medicine, Asthma and Lung Biology, School of Medicine; 3 Department of Environmental Science and Engineering, School of Public Health; 4 Carolina Cardiovascular Biology Center, Department of Medicine, University of North Carolina, Chapel Hill, North Carolina, USA; 5 Harvard School of Public Health, Boston, Massachusetts, USA; 6 Human Studies Division, National Health and Environmental Effects Research Laboratory, ORD, U.S. Environmental Protection Agency, Chapel Hill, North Carolina, USA; 7 Laboratory of Experimental Pathology, National Institute of Environmental Health Sciences, National Institutes of Health, Department of Health and Human Services, Research Triangle Park, North Carolina, USA; 8 Tel Aviv University, Tel Aviv, Israel

**Keywords:** aconitase, air pollution, cardiac gene expression profile, mitochondria, particulate matter, zinc

## Abstract

**Background:**

Exposure to particulate matter (PM) has been associated with increased cardiovascular morbidity; however, causative components are unknown. Zinc is a major element detected at high levels in urban air.

**Objective:**

We investigated the role of PM-associated zinc in cardiac injury.

**Methods:**

We repeatedly exposed 12- to 14-week-old male Wistar Kyoto rats intratracheally (1×/week for 8 or16 weeks) to *a*) saline (control); *b*) PM having no soluble zinc (Mount St. Helens ash, MSH); or *c*) whole-combustion PM suspension containing 14.5 μg/mg of water-soluble zinc at high dose (PM-HD) and *d* ) low dose (PM-LD), *e*) the aqueous fraction of this suspension (14.5 μg/mg of soluble zinc) (PM-L), or *f* ) zinc sulfate (rats exposed for 8 weeks received double the concentration of all PM components of rats exposed for 16 weeks).

**Results:**

Pulmonary inflammation was apparent in all exposure groups when compared with saline (8 weeks > 16 weeks). PM with or without zinc, or with zinc alone caused small increases in focal subepicardial inflammation, degeneration, and fibrosis. Lesions were not detected in controls at 8 weeks but were noted at 16 weeks. We analyzed mitochondrial DNA damage using quantitative polymerase chain reaction and found that all groups except MSH caused varying degrees of damage relative to control. Total cardiac aconitase activity was inhibited in rats receiving soluble zinc. Expression array analysis of heart tissue revealed modest changes in mRNA for genes involved in signaling, ion channels function, oxidative stress, mitochondrial fatty acid metabolism, and cell cycle regulation in zinc but not in MSH-exposed rats.

**Conclusion:**

These results suggest that water-soluble PM-associated zinc may be one of the causal components involved in PM cardiac effects.

Although human exposure to airborne particulate matter (PM) is associated with adverse cardiovascular effects (Pope et al. 2004), the specific causative components or sources have not been identified. Ambient PM is physicochemically heterogeneous and contains significant quantities of metals, including iron, aluminum, silica, zinc, and copper; the levels of these elements vary depending on the geographic location and the local sources (Harrison and Yin 2000; Kodavanti et al. 2005; Ostro et al. 2007; Schwar et al. 1988). Zinc is a ubiquitous PM metal reaching nearly 27 μg/m^3^ airborne concentration in industrial areas of developing countries (Harrison and Yin 2000). Tire- and brakewear may also contribute to the near-road atmospheric concentration of zinc (Adachi and Tainosho 2004; Councell et al. 2004).

Zinc is an essential nutrient required for the maintenance of cell growth, immune maturation, and reproduction and is known to function as an antioxidant via induction of metallothioneins (Maret 2000, 2004; Maret and Sandstead 2006). Although milligram quantities of zinc are ingested daily in foods and with vitamin supplements at slightly higher than physiologic levels, zinc causes cardiovascular and neuronal toxicity (Dineley et al. 2003; Maret 2000, 2006). Although few reports suggest zinc-mediated cardiac toxicity (Evangelou and Kalfakakou 1993; Klevay et al. 1994), the multifaceted effects of zinc on mitochondrial respiration (Ye et al. 2001), calcium homeostasis (Hershfinkel et al. 2001; Maret 2001), sulfur-metal coordination (Maret 2004), intracellular signaling (Samet et al. 2003), myocyte ion channels (Graff et al. 2004), and competition with other essential metals (Klevay 2000; Labbe and Fischer 1984) are well documented and may portend a risk of toxicity.

PM-associated water-soluble metals, including zinc, can be absorbed via the pulmonary vasculature upon deposition, potentially reaching cardiac tissue at high concentrations (Gilmour et al. 2006a; Wallenborn et al. 2007) before it is sequestered in the liver. We recently reported that long-term, episodic inhalation exposure to zinc-enriched combustion particles, similar to some ambient PM (Adamson et al. 2000), caused myocardial injury in the Wistar Kyoto rat (Kodavanti et al. 2003). We further demonstrated that a bolus pulmonary exposure to zinc causes marked changes in cardiac gene expression reflective of the impairment in mitochondrial respiration, cell signaling, Ca^2+^ homeostasis, and ion channel function (Gilmour et al. 2006b). In the present study we have hypothesized that particle-associated, water-soluble zinc is one of the causative PM components responsible for myocardial effects, including oxidative stress and altered cell signaling resulting from protracted exposures. We compared the toxicities of PM with or without zinc, the water-soluble fraction of zinc-containing PM, and soluble zinc alone. Particle exposures with or without zinc increased the incidence of cardiac lesions to a small extent, perhaps as a result of long-term pulmonary inflammation. However, only the soluble zinc, and to some extent zinc-containing PM suspension, or the leachate fraction caused moderate inhibition of cardiac total aconitase activity, mitochondrial DNA damage, and changes in cardiac gene expression. These changes in gene expression are consistent with alterations in cell growth, signaling, mitochondrial fatty acid metabolism, ion channel function, and overall oxidative stress. Thus, PM-associated zinc may be one of the causative components of ambient PM responsible for cardiac effects.

## Materials and Methods

### Animals

Healthy 12- to 14-week-old male Wistar Kyoto (WKY) rats were purchased from Charles River Laboratories (Raleigh, NC, USA). All rats were maintained in an isolated animal room in an Association for Assessment of Laboratory Animal Care–approved animal facility (21 ± 1°C, 50 ± 5% relative humidity, 12-hr light/dark cycle) for 1- to 2-week quarantine and nonexposure periods. The rats were housed in plastic cages with beta chip bedding. All animals received standard PMI5001 rat chow (PMI Nutrition International, St. Louis, MO, USA) and water *ad libitum*. The U.S. Environmental Protection Agency (EPA) Animal Care and Use Committee approved the protocol for use of rats in these studies. Animals were treated humanely and with regard for alleviation of suffering.

### Rationale for selection of PM samples, exposure methods, and concentrations

We have recently reported that a 16-week episodic inhalation exposure of rats to oil combustion PM containing water-soluble zinc caused myocardial injury (Kodavanti et al. 2003). We wanted to determine if cardiac injury was caused by zinc leached from PM or was secondary to pulmonary inflammation that may occur as a result of deposition of PM in the lung. Therefore, we designed a study protocol that used six groups (*n* = 8) of male WKY rats receiving different components of PM (Table 1) for each of two time points (8 and 16 weeks). Group 1 received saline to serve as a control. Group 2 received Mount St. Helens ash (MSH), which does not contain any water-soluble zinc or other metals (McGee et al. 2003). Thus we can delineate any cardiac effect secondary to pulmonary inflammation/injury after deposition of these particles, as these fine-mode particles themselves are not likely to translocate to the heart. Group 3 received whole saline suspension at high dose (PM at high dose level, PM-HD) of the same fugitive oil combustion particle sample used in the previous study (Kodavanti et al. 2002) and contained insoluble components plus water-soluble zinc (14.5 μg/mg of zinc) and also a small amount of water-soluble nickel (3.0 μg/mg). The elemental composition of this PM is comparable to the previously used Ottawa urban PM (Adamson et al. 2000). Group 4 also received this particle sample but at half the dose of group 3 (particulate matter at low dose level, PM-LD). Group 5 received leachable fraction (saline-soluble) of PM-HD devoid of any solid material but containing the soluble components of zinc and nickel (particulate matter, saline leachable fraction, PM-L). Finally, group 6 received saline-solubilized zinc sulfate (zinc) at a concentration of zinc equivalent to group 3 or 5. This design allowed us to test if cardiac injury was due to soluble zinc or secondary to PM-induced pulmonary inflammation.

Because it is technically challenging to use inhalation methodology for exposure of rats to all these fractions concurrently, repeated weekly intratracheal instillations were used. We have shown that the instillation of a similar fly ash can give very similar pulmonary outcome after a single instillation if the instillation and inhalation doses are matched (Costa et al. 2006). Particle dose was based on our previous inhalation study, which used the same PM and led to cardiac injury (Kodavanti et al. 2003). Our previous study included 10 mg/m^3^ of inhaled PM at 6 hr/day for 1 day/week for 16 weeks. The total lung deposition fraction was assumed to be 0.32, and the cumulative lung dose of PM over the 16-week period was calculated to be 5.53 mg for a 300-g rat (18.43 mg/kg body weight). This total deposition amount was divided by 16 for each weekly intratracheal dose in the PM-LD group 4 (1.15 mg/kg). To assure sufficient particle exposure to cause cardiac injury from fractions of the whole particle suspension, group 3 (PM-HD) received double this dose (2.30 mg/kg). The same mass dose of MSH was used for group 2 animals (2.30 mg/kg). A saline-leachable fraction devoid of solid components was prepared from the particle suspension (2.30 mg/kg) and given to group 5 (PM-L). Zinc concentration in this water-soluble fraction of particle was 14.5 μg/mg; therefore, group 6 animals received zinc sulfate solution amounting to 33.4 μg zinc/kg body weight (the amount of water-soluble zinc present in 2.3 mg PM). Rats in the 8-week study received eight weekly instillations but with all the components at double the concentration than those used for 16 weeks. The 8-week exposure was used to determine if cardiac effects can be apparent within a short time, so that future studies can be planned using this timeframe when material is limited. The rationale for doubling the concentration for 8 weeks was to ensure detection of cardiac injury on the basis of the theoretical dose for 16 weeks that was needed for detectable injury in our earlier study (Kodavanti et al. 2003). Because the concentration of the weekly PM doses were the same for the 8-week PM-LD group and 16-week PM-HD groups, we were able to determine the progression of effects with repeated PM exposures.

### Intratracheal instillation

We used WKY rats for this study because this strain has shown lower background cardiac lesions than Sprague-Dawley rats (Kodavanti et al. 2003), although sporadic myopathy is apparent (Kuribayashi 1987). In our previous study, this strain appeared to be specifically sensitive to PM cardiac injury because of low background cardiac spontaneous lesions (Kodavanti et al. 2003). Rats were randomized by body weight into six groups (*n* = 8) for each time point. Each particle sample was suspended in sterile saline at the desired concentration and was mixed continuously for 20 min before use. To prepare the soluble fraction, the PM-HD was centrifuged at 12,000 × *g* for 10 min and the supernatant fluid filtered through a Teflon syringe filter unit (0.25-μm pore size). This filtered fraction was termed as the leachable fraction (PM-L). Similarly, zinc sulfate was dissolved in sterile saline to give desired concentration. All fractions, including saline, were intratracheally instilled once per week at 1 mL/kg under halothane anesthesia (Costa et al. 1986).

### Necropsy, sample collection, and analysis

We selected a 48-hr time point after the last instillation for necropsy to minimize impact of the last intratracheal instillation and maximize detection of subchronic outcomes. Two days after the last instillation, rats were anesthetized with an overdose of sodium pentobarbital (50–100 mg/kg, ip). Blood was drawn, and the heart was removed, blotted dry, weighed, and cut into two mid-longitudinal halves, one for enzyme assay and RNA/DNA isolation and the second for histology. The right ventricle was discarded from the first half and portions of the left ventricle plus septum were snap frozen in liquid nitrogen and retained for enzymes activity analysis and RNA isolation.

The second half of the heart was fixed in 10% neutral-buffered formalin. The first, third, fifth, and eighth consecutive sections were stained with hematoxylin and eosin (H&E) and examined microscopically without prior knowledge of the treatment groups. A more detailed description of histology assessment is provided in the Supplemental Material (http://www.ehponline.org/members/2007/10379/suppl.pdf).

Immediately after removal of the heart, the trachea was cannulated, the left lung tied and the right lung lavaged using Ca^2+^/Mg^2+^-free phosphate-buffered saline (pH 7.4) as previously described (Kodavanti et al., 2002). Aliquots of bronchoalveolar lavage fluid (BALF) were used to determine total cell counts with a Z1 Coulter Counter (Coulter, Inc., Miami, FL, USA).

### RNA isolation

Total heart RNA was isolated from tissues snap frozen in liquid nitrogen using TriReagent (Sigma-Aldrich, St. Louis, MO, USA). RNA was further purified with QIAGEN RNeasy mini columns (QIAGEN, Valencia, CA, USA) and resuspended in 50 μL diethylpyrocarbonate (DEPC)-treated water according to the manufacturer’s protocol. RNA quantity was assessed with an Agilent 2100 Bioanalyzer (Agilent Technologies, Palo Alto, CA, USA). All samples had a 28S/18S ratio ≥ 2.0 and were stored at −80°C before shipment on dry ice to Expression Analysis Inc. (Durham, NC, USA; www.expressionanalysis.com) or their use in real-time polymerase chain reaction.

### Microarray target preparation and hybridization

Expression Analysis Inc. performed RNA target preparation and hybridization to the Affymetrix GeneChip Rat 230A microarray containing 15,923 probe sets and expressed sequence tags (Affymetrix, Inc., Santa Clara, CA, USA) according to the “Affymetrix Technical Manual” (Affymetrix, Inc. 2004). A brief description of the process for cRNA synthesis, hybridization, visualization, and quantification is described elsewhere (Gilmour et al. 2006b). Fluorescent images were detected in a GeneChip Scanner 3000 (Affymetrix), and expression data was extracted using the default setting in the microarray Suite 5.0 software (Affymetrix). For microarray purposes, four biological replicates were collected for each group.

### Real-time PCR

To confirm the Affymetrix gene array data, real-time quantitative PCR was performed for metallothionein-1 (MT-1), and zinc transporter-2 (ZT-2) using heart RNA derived from the 8-week exposure group, essentially as described previously (Gilmour et al. 2006b). Zinc exposure caused a 30% increase in MT-1 mRNA expression over saline control, which is consistent with the data obtained in the microarray in the present study (insignificant in each case). No significant increases were noted in ZT-2 mRNA expression by either method.

### DNA isolation and mitochondrial DNA damage analysis using quantitative PCR (Q-PCR)

We extracted DNA from left ventricular tissues of the 8-week exposure group using a genomic DNA extraction kit (QIAGEN, Chatsworth, VA, USA) according to the protocol supplied with the kit. DNA was quantified using the PicoGreen dsDNA Quantitation kit (Invitrogen Corp., Carlsbad, CA, USA). Q-PCR was conducted using a protocol described previously (Ayala-Torres et al. 2000; Santos et al. 2002), except for the quantification of PCR products, which was performed using Pico-Green dye. The primer sequences used were as follows: for the 12.4-kb nuclear gene, clusterin 5′-AGA CGG GTG AGA CAG CTG CAC CTT TTC-3′ and 5′-CGA GAG CAT CAA GTG CAG GCA TTA GAG-3′; for the 13.4-kb mitochondrial genome, 5′-AAA ATC CCC GCA AAC AAT GAC CAC CC-3′ and 5′-GGC AAT TAA GAG TGG GAT GGA GCC AA-3′; and for the 235-bp mitochondrial fragment, 5′-CCT CCC ATT CAT TAT CGC CGC CCT TGC-3′ and 5′-GTC TGG GTC TCC TAG TAG GTC TGG GAA-3′. DNA lesion frequencies were calculated as described (Mandavilli et al. 2000). Briefly, the amplification of damaged samples (A_D_) was normalized to the amplification of a non-damaged control (A_O_), resulting in a relative amplification ratio. Assuming a random distribution of lesions and using the Poisson probability mass function equation [*f* [*x*!] = *e*^−λ^λ*^x^/x*], where λ = the average lesion frequency for the nondamaged template (i.e., the zero class; *x* = 0), the average lesion per DNA strand was determined as: λ = −*InA_D_/A*_O_.

### Cardiac aconitase activity and protein analysis

Frozen left ventricular tissues (stored at −80°C) were homogenized in ice-cold 10 mM Tris–KCl buffer, pH 7.4, using a polytron homogenizer. Homogenates were centrifuged at 12,000 × *g* for 20 min at 4°C. The supernatants were quick frozen, stored at −80°C, and later analyzed for aconitase activity. Aconitase activity was measured based on the formation of NADPH from NADP^+^ using the Bioxytech Aconitase-340 Assay (Oxis International Inc., Foster City, CA, USA). Total protein content was analyzed using Coomassie Plus Protein Assay kit with bovine serum albumin as a standard (Pierce, Rockford, IL, USA).

### Statistical analysis

We performed statistical analysis of BALF cells and aconitase data using a two-way analysis of variance (ANOVA) with treatment as one factor and time as the other using SigmaStat software, version 3.5 (SPSS Inc., Chicago, IL, USA), whereas one-way ANOVA was performed for cardiac mitochondrial DNA damage data. One control animal at 16 weeks was excluded from the study, as it demonstrated pulmonary complications immediately after the first intratracheal instillation of saline as determined by measurement of breathing parameters. Pairwise comparisons between groups were made using the Fisher least-significant difference (LSD) test. The accepted level of significance was *p* < 0.05.

### Analysis of Affymetrix microarray GeneChip data

Affymetrix CEL data files were imported into R, an open source statistical scripting language (http://www.R-project.org; Ihaka and Gentleman 1996) that was used in conjunction with the Bioconductor project (http://www.bioconductor.org; Gentleman et al. 2004). Normalized values with robust multiarray average (RMA) background correction, quantile normalization, and median polish were calculated with the R/bioconductor package AffylmGUI (Wettenhall et al. 2006). AffylmGUI allows a graphical user interface for the analysis of Affymetrix microarray GeneChips using the LIMMA package (linear modes for microarray data) in R (Smyth 2005). A linear model was fitted to the data and used to average data between replicate arrays and to identify variability between them. Contrasts between groups were used to generate *p*-values, moderated *t* statistics, Empirical Bayes statistics, and M values [log_2_ (ratio)]. The following contrasts were made for this study: MSH/saline, PM-HD/saline, Zn/saline, Zn/MSH. Probe sets with a *p*-value (*p* < 0.05) were judged by the LIMMA package (http://bioconductor.org/packages/2.1/bioc/html/limma.html) to be differentially expressed within group contrasts. This list was further filtered by fold change (> 1.25 and < 0.75).

We deposited the microarray data discussed in the present article into the Gene Expression Omnibus website (GEO; http://www.ncbi.nlm.nih.gov/geo/; Edgar et al. 2002); these data are accessible through GEO series under accession number GSE6541.

The heat map for the differentially expressed gene list was generated using The Institute for Genomic Research Multi-Experiment viewer (TIGR MeV, version 3.0; Saeed et al. 2003). Differentially expressed genes for MSH, PM-HD, and zinc sulfate relative to saline and zinc relative to MSH were identified and grouped manually into functional categories. These genes are listed in the Supplemental Material, Tables 1–3 (http://www.ehponline.org/members/2007/10379/suppl.pdf). Supplemental Material Table 4 depicts fold change in zinc-exposed rats normalized to MSH. The Venn diagram was derived using GeneSpring 7 software (Agilent Technologies) for MSH, PM-HD, and zinc (normalized to saline) to determine commonalities and differences in changed genes between groups.

## Results

### Pulmonary injury as determined by bronchoalveolar lavage

**Figure 1:**
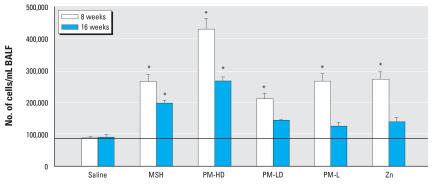
Pulmonary toxicity of soluble and solid PM components as determined by recovery of cells in BALF. Group designations are as follows: saline (control), MSH, PM-HD, PM-LD, PM-L, and Zn (zinc sulfate). Note that 8-week–exposed rats received double the dose of each PM components given to those exposed for 16-weeks. Values represent mean ± SE (*n* = 7–8 rats per group). Horizontal line indicates control levels. *p ≤ 0.05 compared with saline control. Within-group comparison indicated significant differences (*p* ≤ 0.05) at 8 weeks: PM-HD vs. PM-LD; PM-HD vs. PM-L; PM-HD vs. MSH; PM-HD vs. Zn; Zn vs. PM-LD; MSH vs. PM-LD; MSH vs. PM-L and PM-L vs. PM-LD; and at 16 weeks: PM-HD vs. PM-LD, PM-HD vs. PM-L; PM-HD vs. Zn; PM-HD vs. MSH; MSH vs. PM-L; and MSH vs. Zn.

Chronic pulmonary inflammation can influence cardiac physiology. To determine the extent of pulmonary inflammation in each exposed group, we analyzed BALF total cells. Weekly instillations of MSH, PM suspensions (PM-HD and PM-LD), PM-L, and zinc all caused an increase in BALF total cells (Figure 1). The inflammation caused by PM-HD at 8 or 16 weeks was greatest. Exposure to soluble metal-free MSH also increased BALF cells significantly but to a lesser extent than PM-HD. The degree of inflammation was greater in all the 8-week time points postintratracheal challenges compared with the 16-week time point. This was expected, as the 16-week animals received half the concentration of each PM components on a weekly basis compared with the 8-week rats. Although the increases in total cells were apparent at 16 weeks in PM-LD, PM-L, and zinc-exposed rats, the increases were not statistically significant.

### Cardiac histopathology

**Figure 2:**
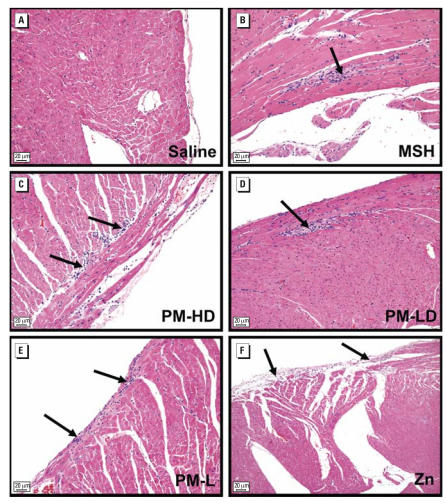
Cardiac histopathological lesions in rats intratracheally exposed to saline or various fractions of PM for 8 consecutive weeks. (*A*) Saline (control), (*B*) MSH, (*C*) PM-HD, (*D*) PM-LD, (*E*) PM-L, and (*F*) Zn. The lesion distribution was focal and not widespread (severity grades 1–2). No specific region appeared more affected over other by PM with or without zinc. Lesions were noted in all exposure groups except for saline controls. Arrows indicate areas of myocardial degeneration and chronic inflammation. In most cases lesions were apparent in subepicardial region; however, no exposure group-related pattern for lesion location was evident. Scale bars = 20 μm.

**Figure 3:**
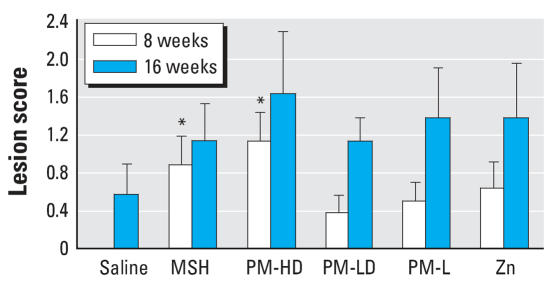
Semiquantitative grading of the extent of lesions within myocardium of rats exposed to different PM fractions. Group designations are as follows: saline (control), MSH, PM-HD, PM-LD, PM-L, PM, and Zn. Pathology severity scores: 0 = none, 1 = minimal, 2 = moderate, 3 = marked, and 4 = severe lesions were employed. Mean severity of lesions was calculated by adding the severity score for all animals within the group and then dividing by total number of animals. Values represent mean ± SE (*n* = 7–8 rats per group). *p ≤ 0.05 compared with saline control.

In the present study the lesions were characterized by foci of myocardial degeneration, inflammation, and fibrosis. These foci were randomly distributed although frequently found at subepicardial or epicardial locations (Figure 2). A careful evaluation of serial sections of myocardial tissues demonstrated no lesions in saline controls at 8 weeks, but two of seven control animals showed mild myocardial degeneration and inflammation at 16 weeks. The photomicrographs for all 16-week exposure groups are depicted in the Supplemental Material, Figure 1 (http://www.ehponline.org/members/2007/10379/suppl.pdf). Generally, exposure of rats to MSH, PM-HD and PM-LD, PM-L, and zinc sulfate all caused small increases in lesion severity relative to saline controls at both time points. The lesion severity was statistically significant in 8-week rats exposed to MSH or PM-HD suspension (Figure 3). However, because of the low incidence of lesions in control rats at 16 weeks, the differences between groups were not statistically significant. Also, because of the limited group size (*n* = 8 for all groups), statistical significance could not be reached across the exposure regimens. Careful evaluation of the location of the lesions in each exposure group revealed no clear distributional differences between groups. Thus, on the basis of a histopathologic evaluation, it was difficult for us to identify the difference in lesion severity between different exposure conditions.

### Cardiac mitochondrial DNA damage

**Figure 4:**
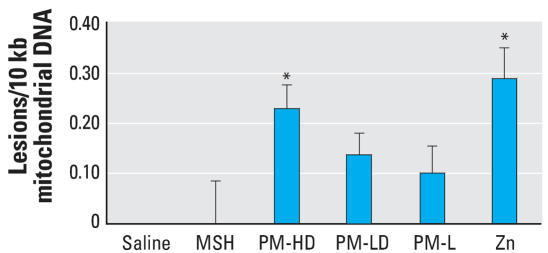
Cardiac mitochondrial DNA damage after eight weekly exposures to solid PM or soluble components in rats. Group designations are as follows: saline (control), MSH, PM-HD, PM-LD, PM-L, and Zn. Note that because of sample-to-sample variation, the only groups that reached statistical significance are Zn and PM-HD, although the trend was consistent in other groups exposed to PM containing water-soluble zinc. Values represent mean ± SE (*n* = 8 rats per group). Note that control values are normalized to zero. * *p* ≤ 0.05 compared with saline control.

The mitochondrial DNA damage in left ventricular tissues from rats exposed for 8 weeks was analyzed using Q-PCR. The rationale of the Q-PCR assay is that the damage in either mitochondrial or nuclear DNA reduces the amplification efficiency of the template, leading to reduction of PCR product with the damaged template. The DNA damage is calculated as discussed in “Materials and Methods.” The results show that the rats exposed to PM-HD and zinc sulfate had significantly increased mitochondrial DNA damage compared with saline (Figure 4). Rats exposed to PM-L also indicated an increase in lesions compared with saline or PM without zinc (MSH), but it was not statistically significant. MSH did not cause mitochondrial DNA damage.

### Cardiac aconitase activity

**Figure 5:**
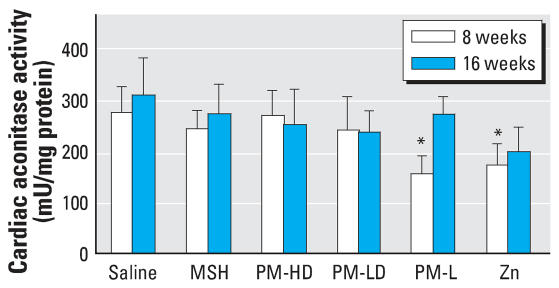
Cardiac tissue total aconitase activity in rats exposed to soluble or solid PM components for 8 or 16 weeks. Group designations are as follows: saline (control), MSH, PM-HD, PM-LD, PL-L, and Zn. Zinc concentration in PM-HD, and PM-L and Zn groups is the same. Note that rats received the same dose of PM or other components for 8 and 16 weeks. Values represent mean ± SE (*n* = 8 rats per group). *p ≤ 0.05 compared with saline control.

Two isoforms of aconitase exist in the cell. One is cytosolic and the other is mitochondrial. The iron–sulfur clusters of both aconitase isoforms are prone to inactivation by oxidative stress (Cairo et al. 2002; Tong and Rouault 2007), and thus their activity analyses have been extensively used to demonstrate oxidative stress. We determined total aconitase activity at both time points in cardiac tissue homogenates, which included cytosolic plus mitochondrial isoform. There was a small but statistically significant inhibition of aconitase activity in rats exposed to zinc and PM-L in the 8-week group. Although a trend of inhibition was apparent, aconitase activity was not significant in other groups compared with saline (Figure 5).

### Cardiac gene expression

**Figure 6:**
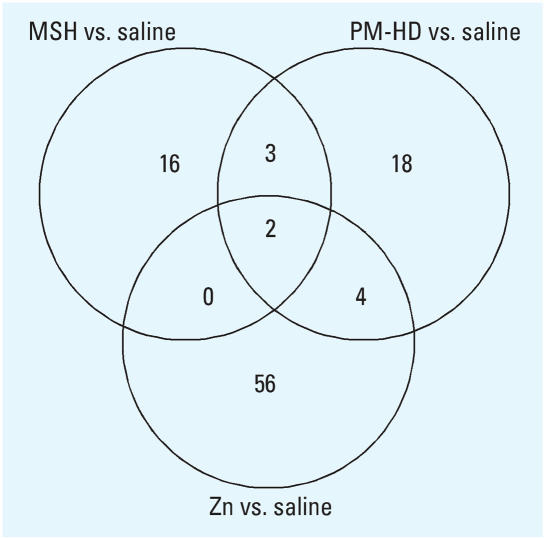
A Venn diagram depicting differences and commonalities in number of genes expressed by given exposure condition. Differentially expressed genes for MSH, PM-HD (whole particle suspension), and zinc sulfate relative to saline were used in developing a Venn diagram. A list of genes for each distinct sections of the Venn diagram is provided in Supplemental Material, Table 5 (http://www.ehponline.org/members/2007/10379/suppl.pdf) .

To understand mechanistic differences between cardiac effects of solid PM without zinc and zinc sulfate, we performed microarray analyses of cardiac tissues from saline, MSH-, PM-HD–, and zinc sulfate–exposed rats at the 8-week time point. Unlike cardiac gene expression changes after high-dose single pulmonary exposure to zinc sulfate (Gilmour et al., 2006b), small changes in a limited number of genes were noted in the present study [Supplemental Material, Tables 1–4 (http://www.ehponline.org/members/2007/10379/suppl.pdf)]. Therefore, false discovery rate correction was not employed to minimize omission of the exposure-related changes. However, we did apply a fold change cutoff for listed genes (> 1.25 and < 0.75). A Venn diagram indicating number of genes commonly or distinctly affected by MSH, PM-HD, and zinc sulfate is given in the Figure 6. Supplemental Material, Table 5 (http://www.ehponline.org/members/2007/10379/suppl.pdf) is a list of genes for each distinct section of the Venn diagram.

**Figure 7:**
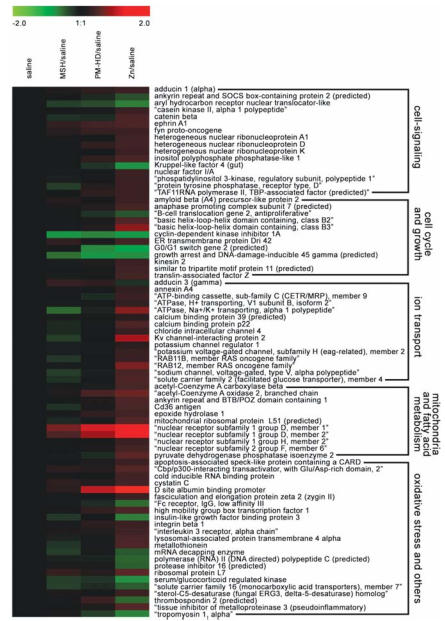
Heatmap showing differential gene expression as fold change intensities from group contrasts with saline control. Red and green color intensities indicate fold change increases and decreases, respectively, in gene expression (expressed as log_2_). Differentially expressed genes for MSH, PM-HD (whole particle suspension), and Zn relative to saline were grouped manually into functional categories.

A very limited number (~ 21 non-EST) of genes showed small increases or decreases with MSH compared with saline controls [Figure 7; Supplemental Material, Table 1 (http://www.ehponline.org/members/2007/10379/suppl.pdf)]. These did not fall into one specific functional category. It should be noted that some of these changes might have occurred by chance alone. Genes commonly affected in MSH and PM-HD containing zinc (Figure 6) included cyclin-dependent kinase inhibitor 1A (decrease), protein tyrosine phosphatase receptor type M (increase), and a gene similar to hypothetical predicted protein CG003 (increase).

The genes in which expression changed in response to PM-HD exposure were very different from those affected by MSH exposure [Figure 7; Supplemental Material, Table 2 (http://www.ehponline.org/members/2007/10379/suppl.pdf)]. Surprisingly, although more non-EST genes were affected (~ 27) by PM-HD compared with changes seen in the MSH group (~ 21), the number of genes showing expression changes within this group was much smaller than the those provoked by zinc exposure (Figure 7). However, some of the genes that changed in PM-HD were the same as those affected by zinc (Figure 6), suggesting that those genes are changed as a result of exposure to zinc.

In Supplemental Material, Table 3 (http://www.ehponline.org/members/2007/10379/suppl.pdf), we list genes that were affected by zinc compared with saline controls. The number of genes affected by zinc was much greater than that changed by MSH or PM-HD suspension (~ 70). These genes were grouped into five functional categories—cell signaling, cell cycle and growth, ion transport, mitochondrial fatty acid metabolism, and oxidative stress/inflammation. Although the fold changes in expression are modest, they are consistent with the known physiologic role of zinc. The small magnitude of change in gene expression in this study may be due to the episodic, lower-dose exposure paradigm rather than by a single high-concentration zinc exposure in our previous study (Gilmour et al. 2006a, 2006b). Also, the 48-hr time point after final instillation used in this study may have allowed reversal of some acute zinc effects.

Our goal was to investigate the role of PM-associated zinc; therefore, we normalized the expression values of zinc sulfate to the MSH, a PM without zinc (McGee et al. 2003). When the zinc values were normalized to MSH, a greater number of genes than those induced by zinc/saline [Supplemental Material, Table 4 (http://www.ehponline.org/members/2007/10379/suppl.pdf)] were found to be differentially expressed, suggesting that the types of expression changes in the heart were very different between soluble zinc and PM or MSH. A variety of genes involved in the acute phase response and oxidative stress were affected, including heat shock 70-kDa protein 1A, and predicted heat shock protein 90-kDa protein 1, which may explain the noted cardiac mitochondrial DNA damage. Metallothionein gene expression was induced, which is consistent with our previous study (Gilmour et al. 2006b), although the increase was small and statistically insignificant (therefore, not included in tables listing genes). Further, similar to the data tabulated in Supplemental Material, Table 3 (http://www.ehponline.org/members/2007/10379/suppl.pdf), a variety of genes involved in cell signaling, cell cycle and growth, ion transport, mitochondrial fatty acid metabolism, and oxidative stress was consistently affected when zinc-induced expression was normalized to MSH (grouped into five functional categories). Only a few genes appeared down-regulated in the hearts of zinc-exposed rats, for example, cyclin D1, fatty acid binding protein 3.

## Discussion

Zinc is a ubiquitous component of ambient PM, often second in abundance to iron. On the basis of our previous study demonstrating cardiac pathology in rats inhaling zinc-containing particles (Kodavanti et al. 2003), we postulated that soluble zinc is one of the causative components of inhaled PM responsible for cardiac effects. To address this hypothesis, we intra-tracheally exposed rats to PM with or without its zinc constituents or to zinc alone once per week for 8 or 16 consecutive weeks. We report here that increased BALF cell numbers and cardiac pathology were evident in all rats exposed for 8 or 16 weeks to PM with or without soluble zinc and to soluble zinc. However, only the rats exposed to soluble zinc or zinc-containing PM demonstrated mitochondrial DNA damage. Aconitase activity was inhibited slightly but significantly in rats exposed to zinc or zinc-containing PM leachate. Furthermore, gene expression profiles of the cardiac tissue demonstrated small but significant changes in expression patterns in rats exposed to zinc but not in rats exposed to MSH, a PM without zinc. These changes were reflective of altered cell signaling, cell cycle and growth, oxidative stress, inflammation, ion channels function, protease/antiprotease balance, and mitochondrial fatty acid metabolism. Thus, it appears that although insoluble PM may induce a small degree of cardiac pathology, perhaps via chronic pulmonary inflammation, soluble zinc may contribute to cardiac injury via its effects on gene expression, and mitochondrial DNA damage.

Cardiac injury from pulmonary PM exposure can occur via systemic endothelial activation and/or lung inflammation (Godleski 2006). Cardiac effects can also occur directly by PM-associated metals (Gilmour et al. 2006b). We cannot determine from our present study the full spectrum of direct effects related to soluble components such as zinc, as cardiac pathology and marked pulmonary inflammation were evident in all groups, including animals exposed to MSH without soluble zinc (McGee et al., 2003). However, distinct gene expression changes, together with small mitochondrial aconitase inhibition and DNA damage, occurred only in zinc-exposed rats. On the basis of this evidence, we postulate that the presence of pulmonary inflammation may lead to pathology in the heart, whereas zinc may affect broader physiologic processes at multiple levels without apparent lesion development. The data support the hypothesis that zinc is responsible, at least partly, for the PM cardiac effects.

In the previous study, we demonstrated that pulmonary exposure to a large bolus of zinc resulted in an approximately 20% increase in circulating zinc at 1 hr after exposure, whereas cardiac effects were manifested at 4- and 24-hr time points, when circulating zinc had returned to control levels (Gilmour et al. 2006b). No net increase in cardiac zinc was noted in that study. Also, the resulting increase in plasma zinc was transient, ultimately with the accumulation of zinc in the liver. Interestingly, at 24 hr, copper and selenium in the liver also increased, suggesting that pulmonary zinc exposure can result in systemic imbalance of other essential metals (Gilmour et al. 2006b). Zinc has been shown to interact with other essential metals in the body and in the heart (Powell et al. 1999). Thus, the cardiac effects might be due to acute transient elevations in circulating zinc, which leads to disturbances in essential metal balance immediately after each instillation. Because zinc is best known to bind to metallothionein protein and a massive induction of this gene has been noted in the lung immediately after instillation of soluble zinc (Gilmour et al. 2006b), it appears that pulmonary zinc might be carried to circulation in its protein bound form (Agte and Nagmote 2004); however, it is not stored in the heart.

At high levels, zinc is known to inhibit mitochondrial respiration in rat liver but not in cardiac tissues (Ye et al. 2001). An increase in circulating zinc, however, has been shown to cause a number of cardiac mitochondrial effects (Gilmour et al. 2006b). It is not known if acute pulmonary zinc exposure inhibits cardiac aconitase activity; however, zinc transfer from metal-lothionein to cardiac mitochondrial aconitase has been noted in mouse heart extracts (Feng et al. 2005). Further, inhibition of cardiac aconitase activity in the present study may involve inactivation of cytosolic and/or mitochondrial isoforms secondary to zinc-induced oxidant production. Aconitase is highly susceptible to inhibition by oxidative stress and has been extensively used as a marker to demonstrate production of free radicals in tissues (Tong and Rouault 2007). The probable involvement of increased oxidant production is also reflected in the increase in mitochondrial DNA damage reported here. The mechanism is not clear because zinc is present in the regulatory domain of numerous proteins within cytosol and mitochondria (Maret 2001, 2004; Sharpley and Hirst 2006). Thus, perturbation of more than one metal–protein coordinations is likely to be involved. Evidence of zinc-induced oxidative stress is seen in the neuronal release of zinc and associated degenerative changes in the brain (Dineley et al. 2003).

Although modest, changes in the gene expression provide important mechanistic insight into potential long-term effects of zinc on the myocardium. Only a few changes occurred in the pattern of cardiac gene expression in MSH-exposed rats, whereas relatively large changes were noted in rats exposed to zinc sulfate. This suggests that a small increases in cardiac lesions may be independent of zinc-specific effect on cardiac gene expression. It is likely that effects on gene expression caused by zinc may not result in chronic cardiac pathology in the present study. It is not clear why exposure of rats to whole PM suspension (PM-HD) containing soluble zinc did not alter as many genes as those altered by zinc. However, it is noteworthy that some of the genes induced by the PM-HD were also induced by zinc, suggesting a zinc-specific effect. It is also possible that the presence of small amounts of nickel (Kodavanti et al. 2002) and also solid PM may interfere with PM-zinc absorption, and metal–metal interactions may limit zinc bioavailability. We have previously noted that the toxicity of one individual metal may be reduced in the presence of other metals (Kodavanti et al. 1997) and interactions of zinc and copper are well documented (Klevay 2000; Powell et al. 1999). The association between PM-induced long-term pulmonary inflammation and increases in the background cardiac lesions need to be further examined.

The changes in the cardiac gene expression pattern from pulmonary exposure to soluble zinc seen in the present study were expected based on the known biological role of zinc in cell growth, metabolism, cell signaling, and mitochondrial respiration (Maret, 2006; Samet et al. 2003; Ye et al. 2001). Although the direct effect of zinc on each of the genes in which expression was altered has not been reported in the literature, the functional grouping of these genes suggests multiple sites of action. For example, numerous genes for proteins, such as kinases and phosphatases, that regulate cell signaling were stimulated, whereas the epidermal growth factor receptor gene was down-regulated. In isolated cells, zinc in protein-free medium is known to modulate phosphorylation of these proteins (Samet et al. 2003). Unlike down-regulation of mRNA expression of phosphatases after one bolus and acute exposure (Gilmour et al. 2006b), in the present study episodic long-term exposure to zinc led to up-regulation of these genes, which may reflect the chronic outcome of multiple episodic exposures.

Excess zinc in isolated cells has been shown to regulate calcium, sodium, and potassium channels (Aedo et al. 2007; Graff et al. 2004; Maret 2001). Rats exposed to zinc but not to MSH showed modulation of genes that form either these ion channels or their regulatory proteins (apparent when zinc data were normalized to MSH). Interestingly, induction was noted, as opposed to the previously reported bidirectional effect, after acute zinc exposure (Gilmour et al. 2006b). This suggests compensatory regulation of cellular homeostasis by activation of ion transport mechanisms. Chronic effects of excess zinc on calcium, potassium, and sodium channels have not been well investigated and may affect cardiac conductance properties. In our subsequent study, heart rates were analyzed in rats exposed to MSH, PM-HD, and zinc (Rowan et al., 2005). Zinc and PM-HD but not MSH caused a small but acute drop in heart rate which was reversible within 1 day. Excess zinc suppresses rat myocyte beat frequency *in vitro* (Graff et al. 2004) and also heart rate in isolated guinea pigs hearts (Evangelou and Kalfakakou 1993). Thus, it is likely that zinc has acute cardiac physiologic effects; however, the long-term effects remain unclear.

Excess zinc modulates mitochondrial respiration in rat liver likely via its effect on electron transport (Kuznetsova et al. 2005). In the present study, zinc exposure did not cause changes in gene expression in components of cardiac electron transport proteins; however, it did increase the expression of genes involved in mitochondrial fatty acid metabolism. It is possible, therefore, that an increase in fatty acid metabolism could cause an increase in free radical production within mitochondria and lead to mitochondrial DNA damage and inhibition of aconitase activity following multiple episodic exposures.

The suppression of gene expression regulating heat shock proteins, stimulation of matrix metalloproteinase inhibitors, changes in hypoxia-regulated genes, and up-regulation of oxidative stress–sensitive genes observed in our study may imply increased free radical production. The excess zinc has been shown to affect these processes in a variety of cell types (Chun et al. 2001; Larbi et al. 2006; Puerta et al. 2006). This zinc-induced oxidative stress response is contrary to its commonly reported cardioprotection and its antioxidant function via metallothionein (Kang 1999; Maret 2006; Satoh et al. 2000).

In summary, we report here that protracted episodic intratracheal exposures to PM with or without soluble zinc resulted in chronic lung inflammation. The inflammatory response in the lung was associated with modest increases in lesion severity in the heart. However, although small, cardiac mitochondrial DNA damage, inhibition of aconitase activity, and changes in cardiac gene expression patterns were observed only in rats exposed to zinc, or (to some extent) zinc containing PM. These findings suggest that long-term inhalation of urban PM containing high levels of zinc may be linked to increased risk of cardiac morbidity.

## Figures and Tables

**Table 1:** Experimental design depicting group designation and weekly exposure concentrations of insoluble (solid) PM mass and soluble zinc sulfate.

Animal group	Test material	Group designation	8 weeks PM-solid instillation (mg/kg/week)	8 weeks Soluble zinc instillation (μg/kg/week)	16 weeks PM-solid instillation (mg/kg/week)	16 weeks Soluble zinc instillation (μg/kg/week)
1	Saline	Saline	0.00	0.00	0.00	0.00
2	Mount. St. Helens ash	MSH	4.60	0.00	2.30	0.00
3	Whole suspension of oil combustion PM at high concentration	PM-HD	4.60	66.8	2.30	33.4
4	Whole suspension of oil combustion PM at low concentration	PM-LD	2.30	33.4	1.15	16.7
5	Saline-leachable fraction of PM high-concentration suspension	PM-L	0.00	66.8	0.00	33.4
6	Zinc sulfate · 7H_2_O	Zn	0.00	66.8	0.00	33.4

Abbreviations: MSH, Mount St. Helens ash; PM-HD, whole particle suspension instilled at high concentration; PM-L, saline-leachable fraction of PM; PM-LD, whole particle suspension instilled at low concentration.
